# Sclerosing Encapsulating Peritonitis: Solving the Diagnosis Challenge of a Rare Entity

**DOI:** 10.1155/2023/4022487

**Published:** 2023-12-27

**Authors:** Anwar Rahali, El Mehdi Aboulfath, Noureddine Njoumi, Mohammed Rebbani, Yasser El Brahmi, Mohammed Elfahssi, Abderrahman Elhjouji, Aziz Zentar, Abdelmounaim Ait Ali

**Affiliations:** Department of Visceral Surgery, Mohammed V Military Teaching Hospital, Faculty of Medicine and Pharmacy, Rabat, Morocco

## Abstract

Sclerosing encapsulating peritonitis (SEP) is an unusual fibroinflammatory disease of the peritoneum marked by the development of a fibrous membrane enveloping generally the small intestines. The knowledge around this subject is not completely understood. And the etiology can be either idiopathic or secondary to several diseases, treatments, and/or medications. We present a case of a 52-year-old man suffering from atypical clinical symptoms including recurrent abdominal ascites and intestinal obstruction. An abdominal computed tomography showed findings typical of SEP. Therefore, the patient benefited from exploratory laparotomy, which confirmed the diagnosis of idiopathic SEP. Postoperatively, he again had an episode of bowel obstruction, but this was controlled with steroids. Diagnosis of SEP is a real challenge to surgeons, gastroenterologists, and radiologists. And imagery is very helpful to make the diagnosis. Consequently, it is imperative that all hospital practitioners should distinguish between this lesion and other etiology of acute peritonitis.

## 1. Introduction

Sclerosing encapsulating peritonitis (SEP) is a rare condition of mechanical bowel obstruction and acute surgical abdomen. It is characterized by a fibrocollagenous membrane encasing the intestines [1]. This uncommon clinical entity might be secondary to an underlying cause, or it can occur without any preexisting risk factor [2]. We report a case of an adult patient who presented with features of acute intestinal obstruction on a background of recurrent abdominal ascites for the past 3 months. He was managed surgically by excision of sclerosing fibrinous membrane followed by a short course of steroids for his partial bowel obstruction.

## 2. Case Presentation

A 52-year-old man developed acute onset of perpetual widespread abdominal pain and intermittent episodes of postprandial vomiting and nausea and presented to the emergency department 12 hours after the beginning of symptoms. He was a tobacco user (twenty six pack-years) and former excessive alcohol consumer. There was a medical history of recurrent abdominal ascites during the past 3 months which was misdiagnosed as abdominal tuberculosis based on presumptive elements including the following: the patient hails from an area with a high prevalence of tuberculosis, chronic abdominal pain, unexplained weight, and appetite loss. He took antituberculosis therapy for 3 months without clinical improvement. A physical examination revealed marked widespread abdominal tenderness accompanied by abdominal distension without muscular defense.

His baseline workup showed increased inflammatory marker levels such as plasma CRP.

**Figure 1:**
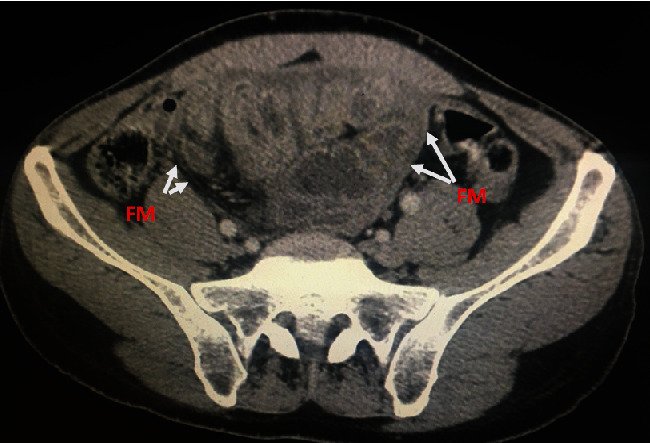
CT imaging dilated small bowel loops, encapsulated by a fibrous membrane (FM) and trapped in the right peritoneal cavity.

**Figure 2:**
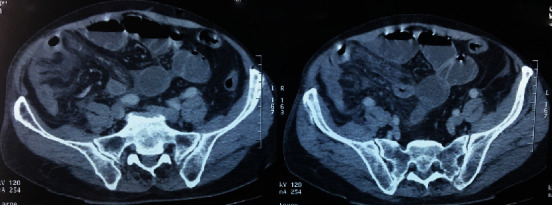
Air-fluid levels in favor of occlusion of the small intestine upstream of the fibrous sac.

An abdominal contrast-enhanced CT showed distended thickened bowel loops which were contained in a fibrocollagenous membrane pushed in the middle of the abdominal cavity; the findings were suggestive of sclerosing encapsulating peritonitis (Figures 1 and 2).

**Figure 3:**
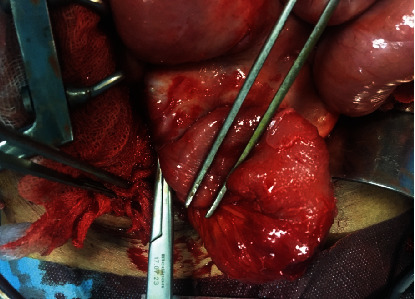
Intraoperative image of the thin membrane encapsulating small bowel.

**Figure 4:**
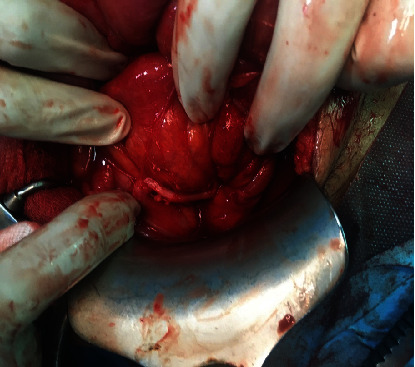
Intraoperative image showing a normal appendix.

Hence, a decision was made for a diagnostic laparotomy in which sclerosing fibrinous membrane encapsulating small intestine was excised, and whole bowels were freed until the ileocecal junction without any injury (Figure 3). The rest of the digestive tract appeared grossly unremarkable especially the appendix (Figure 4).

The patient was discharged on the fifth postoperative day after complete oral feeding resumption. However, he was again admitted on the second day after his discharge with subocclusive intestinal syndrome. He was kept on parenteral nutrition, and he had a short course of methylprednisolone for 5 days at the rate of 120 mg per day. He responded very well, and he was discharged in a stable condition after transit resumption.

After 21 days, the patient appeared asymptomatic. There were no abnormal findings on CT, and he was referred to internal medicine consultation for additional care.

## 3. Discussion

SEP was first reported by the authors of [2] who invented the term “abdominal cocoon syndrome” when detailing the exploratory laparotomy of the condition in 1907. It is an unusual inflammatory lesion that results in the formation of a fibrocollagenous membrane enveloping the abdominal visceral organs [1, 2]. It can be classified as either primary or secondary, commonly caused by things such as abdominal tuberculosis, peritoneal dialysis, intraperitoneal chemotherapy, sarcoidosis, liver cirrhosis, liver transplantation, endometriosis, prior abdominal surgery, certain medications, and malignancy [3].

The pathophysiology of SEP has been reported as four different stages: a presclerosing encapsulating peritonitis, an inflammatory phase, a progressive phase, and a fibrotic phase. The fibrotic phase has been affiliated with the formation of a thick fibrotic membrane encasing the abdominal content and causing small bowel obstruction as in our case [4].

Clinical symptoms are nonspecific and include abdominal pain, abdominal fullness, nausea, vomiting, anorexia, pyrexia, change in bowel habit, and intestinal obstruction, which makes preoperative diagnosis difficult [4]. However, with the arrival of latest imaging technology like high-resolution CT or magnetic resonance imaging, distinguishing SEP from other peritonitis seems possible [5]. As in the present case, the imaging appearance of SEP is visualized as the central accumulation of the small bowel encased by a thin membrane and loculated fluid collections. Other lesions such as ascites, intestinal obstruction, abdominal calcifications, and lymphadenopathy may be present [6].

Some other diseases that may give similar clinical and radiological presentation of SEP include peritoneal tuberculosis, peritoneal mesothelioma or pseudomyxoma, and congenital peritoneal encapsulation [7, 8, 9].

Treatment is determined on a case-by-case basis. Patients with minimal abdominal symptoms are managed conservatively with bowel rest, nasogastric decompression, and parenteral nutritional support [10]. Patients with severe symptoms of small bowel obstruction and who do not respond to conservative management may be candidates for surgical treatment involving excision of the fibrous membrane plus adhesiolysis [11]. In the present case, surgical management was pursued due to both intestinal obstruction and elevated levels of inflammatory markers. Laparoscopy has both diagnostic and therapeutic purposes; technically, it is a real therapeutic challenge in patients with an advanced abdominal cocoon [12].

The most important element predicting postsurgical outcomes of SEP is peritoneal alteration, and the prognosis depends on the cause of the condition, with reported mortality rates as high as 69%, and it occurs generally within a few weeks or months of the interventions [13].

## 4. Conclusion

Despite the rarity of SEP, it is important that surgeons must be conscious of its existence because, clinically and radiologically, it may be confounded with other peritonitis. Consequently, diagnosis of this nonspecific inflammatory condition is a real challenge to all hospital practitioners.

## Abbreviations

SEP: Sclerosing encapsulating peritonitis
CT: Computed tomography
FM: Fibrous membrane.
